# Cervical pedicle morphometry in a Latin American population: A Brazilian study

**DOI:** 10.1097/MD.0000000000003947

**Published:** 2016-06-24

**Authors:** Carlos Fernando Herrero, Anderson Luis do Nascimento, Daniel Augusto Carvalho Maranho, Narcélio Mendes Ferreira-Filho, Carolina Pinto Nogueira, Marcello Henrique Nogueira-Barbosa, Helton Luis Aparecido Defino

**Affiliations:** a Department of Biomechanics, Medicine, and Rehabilitation of the Locomotor System; b Ribeirão Preto Medical School; c Radiology Division of the Internal Medicine Department, Ribeirão Preto Medical School, São Paulo, Brazil.

**Keywords:** anatomy, cervical spine, computed tomography, pedicle

## Abstract

The goal of this study was to conduct a detailed computed tomography (CT) assessment in the Brazilian population of the screw starting point, trajectory, and dimensions of pedicle in the cervical spine.

Two hundred consecutive patients were retrospectively evaluated using cervical spine CT, with imaging reconstruction of each cervical vertebrae in the axial plane with 2 mm, and in sagittal reconstructions with 3 mm. Parameters in axial plane included the pedicle width (PW), pedicle axis length (PAL), pedicle transverse angle (PTA), and the distance from the entry point to the point between the lamina and spinous process (DEP). Measurements in the sagittal plane involved the pedicle height (PH) and the pedicle sagittal angle (PSA).

The mean PW and PH were smaller in females than in males in all cervical vertebrae, but there were no significant differences of PTA among genders. PSA ranged from 15.2° to 23.7°. Mean values of PAL and DEP had a tendency to decrease from the proximal to distal cervical vertebrae. PW was <4 mm in 7.5% of men (C3) and 25% of women (C3), and <4.5 mm in 20% (C3 male) and 66% (C3 female). The intra- and inter-observer reliability were very good for the tomographic measurement of PW, and good for PH. For PAL, the intraobserver reliability was good, but the interobserver reliability varied from moderate to good. Considering PTA and PSA, the intraobserver reliability was good, but the interobserver reliability moderate for PTA and poor or fair for PSA. DEP measurements showed poor intraobserver reliability, and poor or moderate interobserver reliability.

Our results presented similar trend of previous studies, but the frequency of patients with PW <4.5 mm in our population is higher, suggesting an increased risk during the attempting of transpedicular screw technique.

## 1 Introduction

Posterior fixation of the cervical spine is the treatment of choice for several disorders as infection, tumor, trauma, and degenerative disease, depending on instability and anatomical features. Several techniques have been described for treatment including interspinous wiring, laminar hooks, lateral mass screw fixation, and more recently, transpedicular screw fixation.^[1,2]^ In the cervical pedicle screw fixation, a screw is inserted from the lateral mass to the vertebral body, passing all inside the cervical pedicle that acts as a bony bridge. For each side, a rod is used to connect the screws and stabilize the cervical segment. Furthermore, biomechanical studies have shown that cervical pedicle screw provides greater stability than other posterior cervical fixation techniques.^[3–6]^

In 1994, Albumi et al^[7]^ first described the transpedicular screw fixation. Since then, several clinical and imaging studies have reported anatomic features of the cervical pedicle, and the feasibility of transpedicular screw technique.^[8–10]^ However, the method is not universally used due to its challenges and risks.^[11,12]^ Insertion of the cervical pedicle screw may be difficult and risky, because of the reduced dimensions of pedicles and potential of damaging the surrounding neurovascular structures.^[7,11,12]^ Therefore, variability of the ideal trajectory and small dimensions demand a rigorous preoperative evaluation of the pedicle morphometry.^[13–15]^

Despite the cervical pedicle surgical anatomy was well documented in North America, Asia, and Europe,^[9,13,14]^ the anatomical parameters of the cervical spine pedicles may vary among different regions, and there are limited studies about a Latin American population, detailing bony landmarks and parameters to establish the ideal trajectory and feasibility of the cervical transpedicular screw technique. Our hypothesis was that morphometric parameters of the cervical pedicle of a Brazilian population would differ from previously reported data. The objective of our study was to conduct a detailed computed tomography (CT) assessment of screw starting point, trajectory, and dimensions of the cervical pedicle.

## 2 Methods

The local Ethics Committee on Research, Clinics Hospital, Ribeirão Preto Medical School at the University of São Paulo approved the study protocol. Two hundred consecutive patients submitted to cervical spine CT in our institution were retrospectively evaluated. We studied 100 males and 100 females. The mean age of the men was 38 ± 16 years (range 18–83 years) and the average age of the women was 43 ± 18 years (range 18–85 years). Subjects with evidence of severe degenerative, congenital, traumatic, infectious, or neoplastic spine disorders were excluded from the study.

Cervical spine CT scans were performed using the Brilliance CT Big Bore 16-slice (Philips Healthcare, Cleveland, OH) with the standard clinical protocol of the institution. For each cervical vertebra, axial CT reconstruction images with 2 mm thickness and sagittal and coronal reconstructions with 3 mm thickness were available. Axial and sagittal images of the cervical pedicles from the third (C3) to the seventh cervical vertebra (C7) were selected. The parameters were evaluated in the sagittal and axial planes. The axial parameters included the pedicle width (PW), the pedicle axis length (PAL), the pedicle transverse angle (PTA), and the distance from the entry point to the point between the lamina and spinous process (DEP). The intersection of the pedicle longitudinal axis and the posterior cortex was considered the screw entry point. The sagittal measurements involved the pedicle height (PH) and the pedicle sagittal angle (PSA). The percentage of pedicles with the width under 4, 4.5, 5, 6, and 7 mm was calculated (Tables 4 and 5).

**Table 4:**
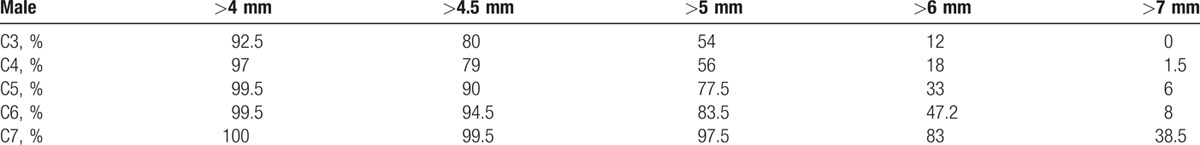
Pedicle width distribution in the male sample.

**Table 5:**
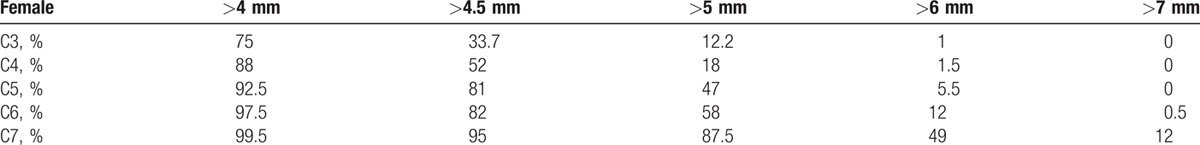
Pedicle width distribution in the female sample.

Two blinded observers independently measured the tomographic parameters using the OsiriX MD Imaging Software, version 7.0.2 (Pixmeo SARL, Bernex, Switzerland). The first observer performed an additional blinded measurement with a 2-month interval between measurements, for estimation of intraobserver reliability.

**Figure 1:**
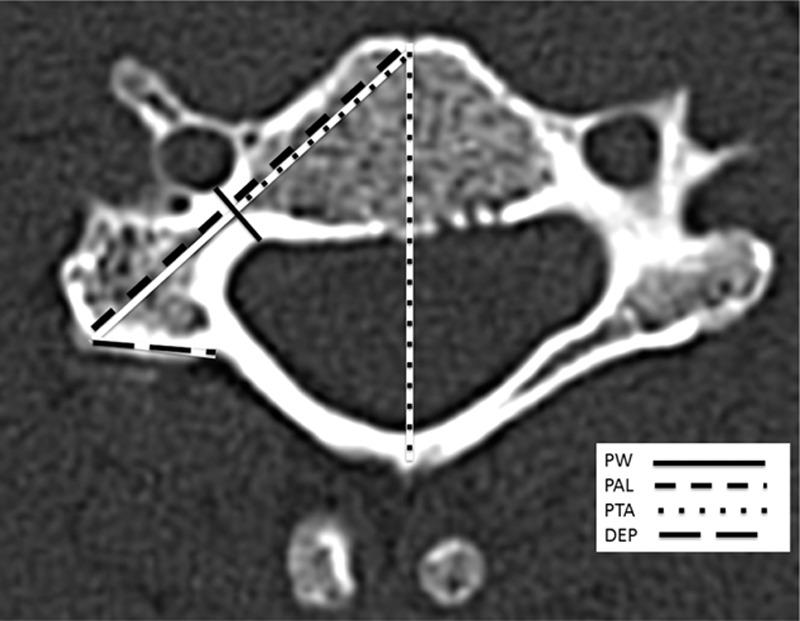
Axial computed tomography reconstruction.

**Figure 2:**
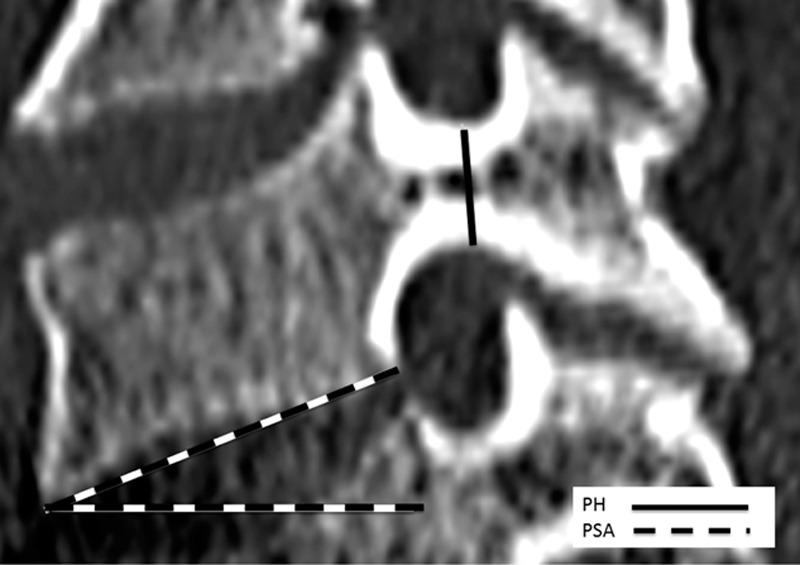
Sagittal computed tomography reconstruction.

The list of the terminology of all parameters that were measured with their abbreviation and description is summarized in Tables 1 and 2. The measurement method is shown in Figs. 1 and 2. Linear parameters were measured in millimeters (±1 mm), and angular parameters were estimated to 1/10th of a degree. Averages and standard deviations were calculated for all pedicle dimensions.

**Table 1:**
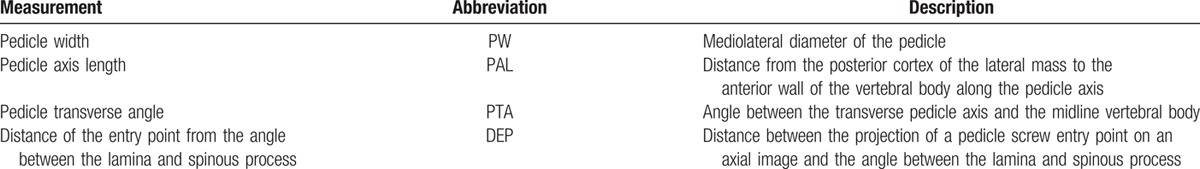
Parameters measured on axial computed tomography images.

**Table 2:**
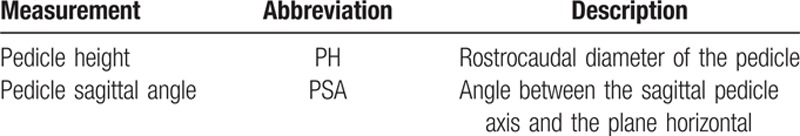
Parameters measured on sagittal computed tomography images.

The usual challenge to transpose diagnostic imaging data into surgically useful information involves the difficulty of depicting 3-dimensional structures, such as the cervical pedicle, in 2 dimensions. To obtain the most pertinent clinically applicable data from the CT images, multiplanar (axial, sagittal, and coronal) evaluation was simultaneously used to measure all the parameters.

### 2.1 Statistical analysis

CT measurements were calculated as means and standard deviations. A total of 2000 pedicles including cervical vertebrae from C3 to C7 were examined. The means and standard deviations of the linear and angular parameters were calculated at each level for male and female patients separately (Table 3). Twelve thousand tests were employed to determine the difference of all dimensional and angular parameters between genders at the same level. Averages and standard deviations were calculated for the pedicle dimensions, and measured values were compared using Student *t* test.

**Table 3:**
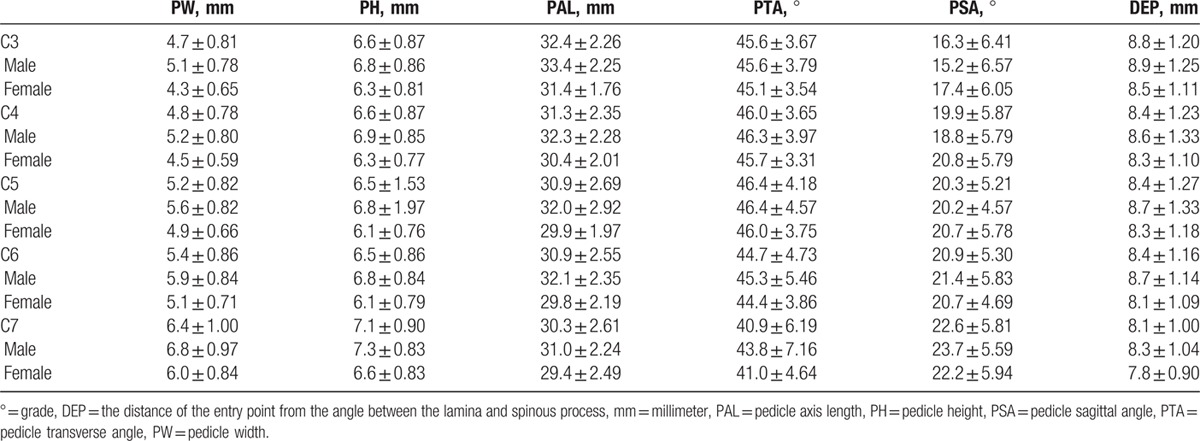
Summary of linear and angular cervical pedicular parameters.

Intra- and inter-observer reliability was estimated using intraclass correlation coefficients (ICC) for the tomographic measurements. A 2-way mixed-effects model with consistency of agreement was applied. Poor reliability is suggested when values are between 0 and 0.20, fair reliability for values from 0.21 to 0.40, moderate reliability for values from 0.41 to 0.60, substantial or good reliability for values from 0.61 to 0.80, and nearly perfect or very good reliability for values from 0.81 to 1.0.^[16]^ We used a significance level of 5% and Stata 14 software for the statistical analysis.

## 3 Results

### 3.1 Axial parameters

**Figure 3:**
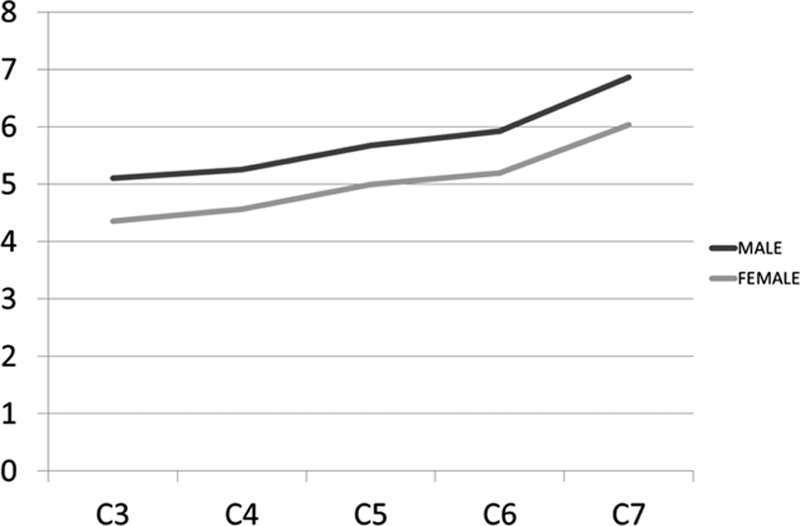
Graph showing the mean pedicle widths in males and females.

The general PW ranged from 4.3 to 6.8 mm (Table 3). The smallest mean PW was found at C3 in both females (4.3 mm) and males (5.1 mm), while the largest mean PW was at C7 in both females (6.0 mm) and males (6.8 mm). There was a tendency toward increasing PW as 1 advances distally in the cervical spine (Fig. 3). The mean PW was smaller in females than in males at all levels, and this difference was very highly significant at all levels (*P* < 0.001). The percentage of individuals with PW <4 mm is 7.5% (C3 male) and 25% (C3 female) and the percentage of individuals with PW <4.5 mm is 20% (C3 male) and 66% (C3 female).

**Figure 4:**
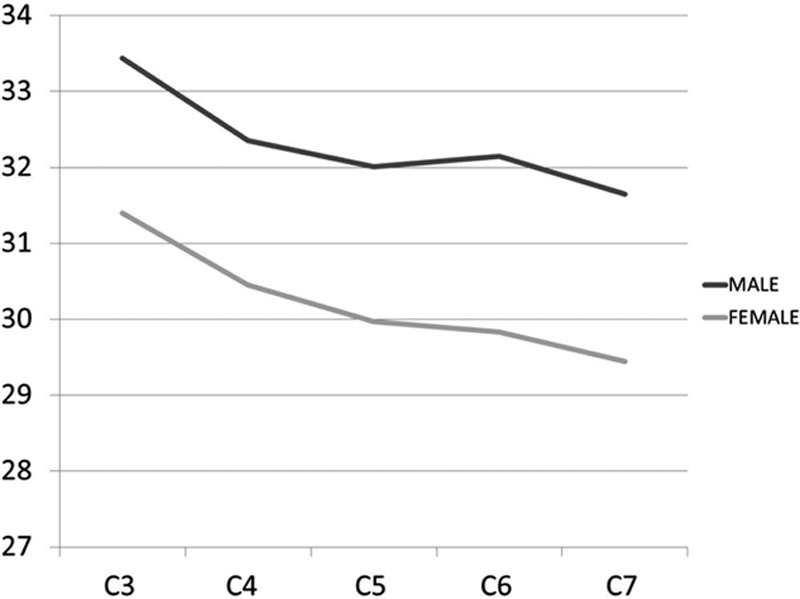
Graph showing the mean pedicle axial lengths in males and females.

The overall mean PAL ranged from 29.4 to 33.4 mm (Table 3). The smallest mean PAL was found at C7 in both females (29.4 mm) and males (31.0 mm), while the largest mean value was found at C3 in both females (31.4 mm) and males (33.4 mm). The mean axial length was smaller in females than in males at all levels, and this difference was very highly significant at all levels (*P* < 0.001). There was a tendency toward decreasing PAL as 1 advances distally in the cervical spine (Fig. 4).

**Figure 5:**
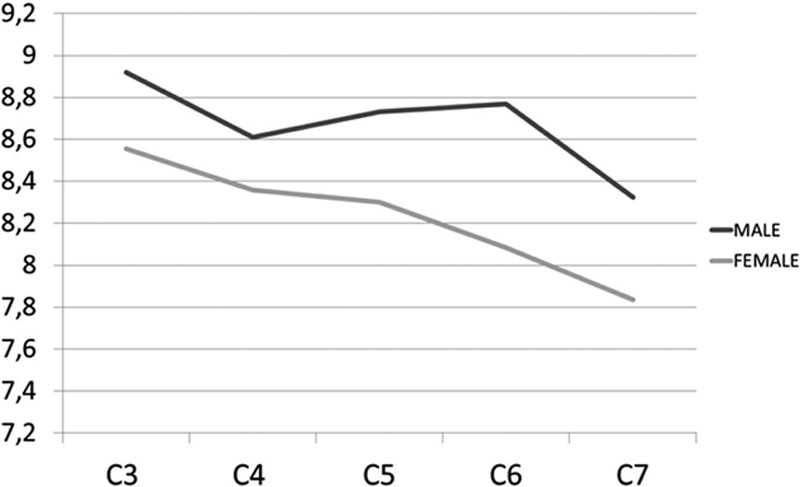
Graph showing mean distances of the entry point from the angle between the lamina and spinous process in males and females.

The mean DEP ranged from 7.8 to 8.9 mm (Table 3). The smallest mean DEP was found at C7 in both females (7.8 mm) and males (8.3 mm). The largest mean DEP was at C3 in both females (8.5 mm) and males (8.9 mm). There was a tendency toward decreasing DEP as 1 advances distally in the cervical spine (Fig. 5). There was no statistical difference among genders.

**Figure 6:**
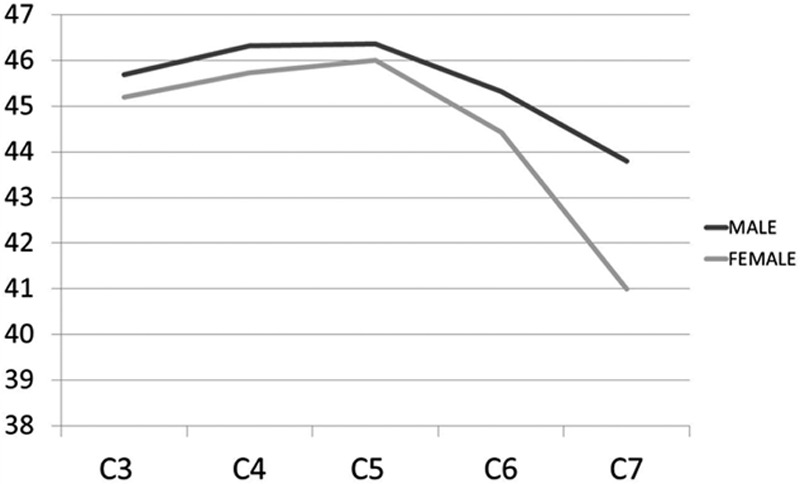
Graph showing mean pedicle transverse angles in males and females.

The overall mean PTA ranged from 41° to 46.3° (Table 3). The smallest mean PTA was found at C7 in both females (41°) and males (43.8°), while the largest mean PTA was at C5 in both females (46.0°) and males (46.4°). There was no statistically significant difference between genders at any level (Fig. 6).

### 3.2 Sagittal parameters

**Figure 7:**
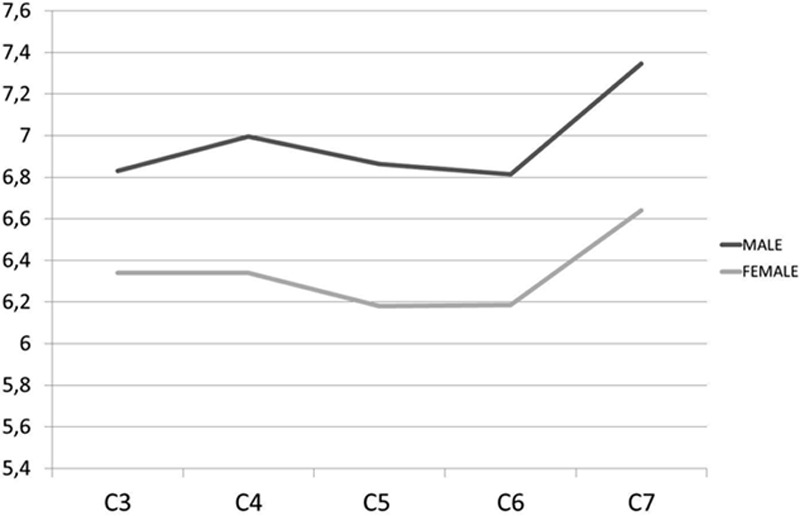
Graph showing mean pedicle heights in males and females.

The general mean PH ranged from 6.1 to 7.3 mm (Table 3). The smallest mean PH was found at C5 and C6 in females (6.1 mm) and C3, C5, and C6 in males (6.8 mm), while the largest mean PH was at C7 in both females (6.6 mm) and males (7.3 mm). The mean PH was smaller in females than in males at all levels, with significant difference (*P* < 0.001). There was no gradual increase in mean PH advancing caudally in the cervical spine (Fig. 7).

**Figure 8:**
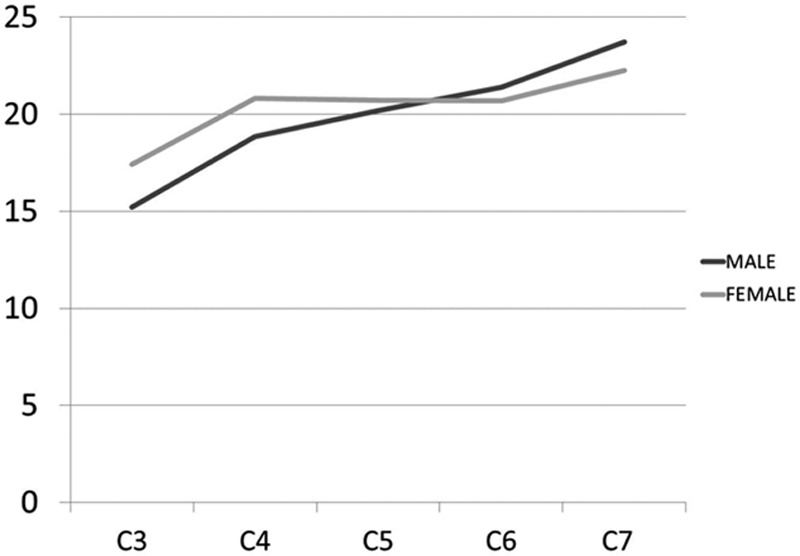
Graph showing mean pedicle sagittal angles in males and females.

Mean PSA ranged from 15.2° to 23.7° (Table 3). The smallest mean PSA was found at C3 in both females (17.4°) and males (15.2°). The largest mean PSA was found at C7 in both females (22.2°) and males (23.7°) (Fig. 8). There was statistical difference among genders in the C3 and C4 at both sides and in C7 and the right side.

### 3.3 Intra- and inter-observer reliability

The intra- and inter-observer reliability were very good for the tomographic measurement of PW (ICC intraobserver 0.92; ICC interobserver 0.88 and 0.89), and good for PH (ICC intraobserver 0.79; ICC interobserver 0.66 and 0.72). For PAL, the intraobserver reliability was good (ICC 0.76), but the interobserver reliability varied from moderate to good (ICC 0.57 and 0.61). Considering PTA and PSA, the intraobserver reliability was good (ICC 0.68 and 0.67, respectively), but the interobserver reliability moderate for PTA (ICC 0.42 and 0.54) and poor or fair for PSA (0.16 and 0.37). DEP measurements showed poor intraobserver reliability (ICC 0.14), and poor or moderate interobserver reliability (ICC 0.20 and 0.49) (Table 6).

**Table 6:**
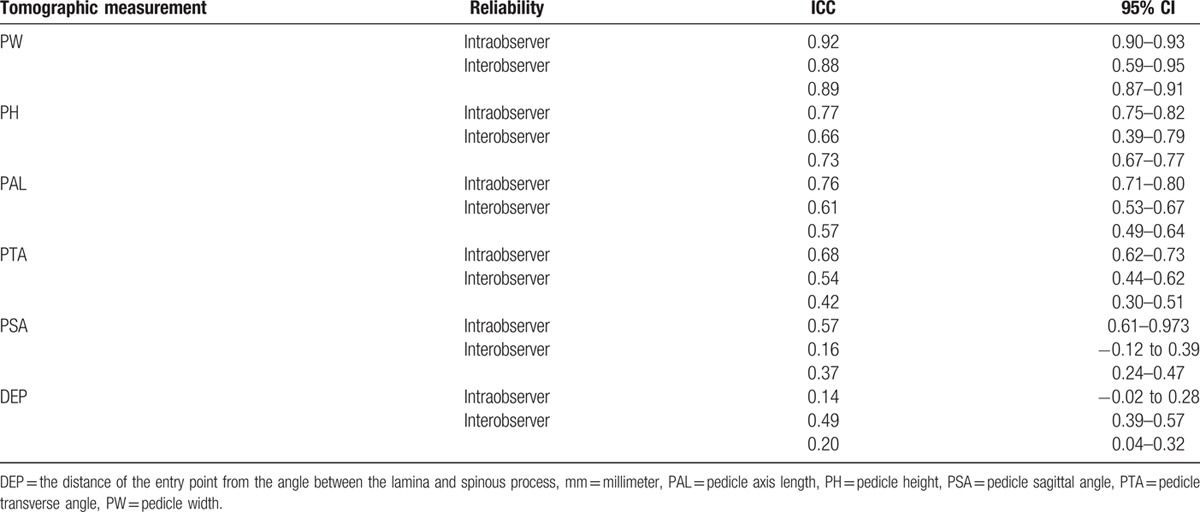
Intraclass correlation coefficients (ICC) with the respective 95% confidence interval (95% CI) for intra- and inter-observer reliability estimation.

## 4 Discussion

Our results demonstrate that anatomical morphometric features of the cervical pedicles of the studied population are similar to previously reported data. The majority of existing studies on cervical transpedicular screw fixation relied on the assessment of a limited number of patients when compared to our study.^[14,15,17]^ To the best of our knowledge, this is the first time that cervical pedicle dimensions of a Latin American population are measured and analyzed regarding gender and spinal level.

Because of the anatomy of cervical spine pedicles in our population may differ from others, the pedicle parameters must be studied to verify if the transpedicular screw fixation can be performed and accepted as a standard procedure. As many other morphometric anatomical differences found in different races and ethnicities,^[13,14,18]^ we believe that data extracted from previous studies should be used with caution because they might not apply universally.

Despite several clinical, radiological, and cadaveric specimen studies regarding cervical pedicle screw placement, there is still controversy regarding the ideal trajectories and entry points.^[7,19–21]^ Transpedicular screw fixation in the cervical spine has been proved to be biomechanically more stable than other posterior fixation techniques, for patients with selected disorders.^[1,5,22,23]^ However, justification from the biomechanical standpoint has to outweigh the potential risk of damaging surroundings neurovascular structures such as spinal cord, nerve roots, and vertebral artery.^[11,12,24]^

Previous CT studies of cervical pedicle dimensions and parameters have included European, American and Asian populations. Despite increasing application of posterior fixations for cervical spinal diseases, distinct morphometric features may exist in the treatment among several ethnic groups. Chazono et al^[13]^ reviewed published data about pedicle dimensions and parameters of the cervical spine through the English literature and failed to identify significant ethnic disparities in pedicle dimensions. The smallest mean PW found was in C4 Asian male with 5.1 mm and C4 European female with 4.1 mm while the largest mean PW found was in C7 Asian male with 7.7 mm and C7 Asian female with 7.0 mm. Our results revealed the smallest mean PW in C3 male with 5.1 mm and C3 female with 4.3 mm, while we found the largest mean PW in C7 male with 6.8 mm and C7 female with 6.0 mm. Our measurements of pedicle dimensions showed that the parameters from C3 to C7 were similar to those previously reported, with similar trend.^[18]^ The PW and PH showed an increase from C3 to C7 in females and males. There was a tendency toward decreasing PAL as 1 advances distally in the cervical spine. On the other hand, we noticed an increase of the PSA as 1 move from caudal to cranial, both in males and females. In agreement with previous studies, we found no significant differences between measurements in males and females. The intra- and inter-observer reliability were very good for the tomographic measurement of PW (ICC intraobserver 0.92; ICC interobserver 0.88 and 0.89), and good for PH (ICC intraobserver 0.79; ICC interobserver 0.66 and 0.72).

Another possible concern regarding cervical pedicle screw fixation might be a too steep angle of insertion that is likely to occur when a cervical pedicle screw is inserted through a standard approach to the cervical spine. Besides that muscle may deviate instruments toward the sagittal plane. Thus larger insertion angles should be avoided through a standard posterior approach since this would demand an extensive unacceptable retraction of the nuchal muscles. Our study did not support previous findings regarding PTA, because our results showed different values, ranging from 41° to 46.3° with the largest mean PTA at C5 and the smallest mean PTA of at C7.^[18]^ We could not observe significant differences in PTA among genders, and the interobserver reliability was moderate for PTA (ICC 0.42 and 0.54) and poor or fair for PSA. Hereafter, preoperative CT assessment of PTA is essential to establish the safe and ideal trajectory for pedicle screw insertion. We do not recommend the use of standardized angles for transpedicular screw technique.

The entry points and trajectories must be based on the ideal pedicle trajectory, which is the line passing through the center of the pedicle in all 3 planes. Many studies reported methods to increase the accuracy of cervical pedicle insertion, including the use of topographic landmarks,^[19]^ measurements of several dimensions and parameters, and surgical techniques or devices.^[10,19,21,25,26]^ Several attempts to define the ideal entry point of the cervical pedicle screw have been reported; however, this is still a source of debate.^[27]^ The first attempt is attributed to Albumi et al^[7]^ who affirmed that the entry point should be somewhat lateral to the center of the articular mass and near to the inferior articular process of the superior vertebra. Because this description is subjective and the landmarks spatial location may vary, the reproducibility is not ideal, and Lee et al^[27]^ suggested 3 landmarks as reference points; the lateral notch, the superior ridge, and the center of the lateral mass. Our study tried to introduce a new landmark as reference point, considering the entry point as the intersection of the pedicle axis and the posterior cortex, then measuring the distance of the entry point from the angle between the lamina and the spinous process (DEP). The poor or moderate intra- and inter-observer reliability observed in our findings reinforce the difficulty to define a rule to identify the ideal entry point. Nevertheless, our results showed a tendency toward decreasing DEP as 1 advances distally in the cervical spine. The mean DEP ranged from 7.8 mm (C7 females) to 8.9 mm (C3 males) and, despite those values were not statistically different, the failure in assessing the correct entry point could lead to serious screw misplacement, and the risk of cortical breakage would increase.

The minimum cervical PW of 4.5 mm was proposed by Yusof et al^[15]^ and has been used as a reference value in other studies. The critical value of 3.5 mm transpedicular screw fixation was proposed considering the 0.5 mm bony wall laterally and medially as measure of safety, to avoid catastrophic damage to the surrounding neurovascular structures.^[7,11,12]^ According to previous studies, 4.2% to 16.7% of the males and 7.1% to 56.3% of the females had pedicles unsuitable for a 3.5 mm screw fixation.^[15]^ The smallest values were found with PW, and C3 presented the lowest mean diameter in males (5.1 mm) and females (4.3 mm). Our study showed that 4.0 and 4.5 mm pedicle screws are too big and, therefore, are unsuitable for routine cervical spine instrumentation in our population. When considering a regular 3.5 mm screw size, the percentages of individuals with PW inappropriate for transpedicular screw technique are 7.5% (C3 male) and 25% (C3 female) in patients with PW smaller than 4 mm, and 20% (C3 male) and 66.3% (C3 female) in patients with PW smaller than 4.5 mm.

This study has limitations that deserve mention. First, this is a retrospective study. The second limitation is the absence of a clinical assessment of the transpedicular screw technique in our study, once the use of the method could emphasize the importance of a preoperative evaluation.

Considering the proportion of individuals detected in our analysis with cervical PW incompatible with a 3.5-mm screw size, the preoperative evaluation of the cervical pedicle anatomic parameters, primarily the PW, should be performed to evaluate safety and feasibility of transpedicular screw technique. We recommend that spine surgeons must be aware and prepared to use alternative methods, such the lateral mass screw technique.

## 5 Conclusion

Despite our results are similar to previously reported studies, the frequency of patients with PW <4.5 mm in our population is higher, suggesting an increased risk during the attempting of transpedicular screw technique. Our results reinforced the necessity of adequate preoperative planning or intraoperative sectional imaging.

## Acknowledgments

The authors acknowledge FAPESP (2014/02752-2) for funding support of the project.
